# From the bench to practice – Field integration of community-based services for older citizens with different levels of functional limitation across European Regions

**DOI:** 10.37825/2239-9747.1020

**Published:** 2020-10-01

**Authors:** G Liotta, R Roller-Wirnsberger, G Iaccarino, E Goossens, C Tziraki, P Obbia, S Pais, F Cacciatore, V Zavagli, F Schena, A Vinci, G D’Amico, E Terraciano, S Gentili, S Lindner, M Illario, the participants to the working groups

**Affiliations:** 1 University of Rome “Tor Vergata”, Department of Biomedicine and Prevention, Rome, Italy; 2 Medical University of Graz, Department of Internal Medicine, Graz, Austria; 3 University of Naples “Federico II”, Department of Advanced Biomedical Sciences, Naples, Italy; 4 Center for Gastrology, Leuven, Belgium; 5 Research Institute, Melabev and Hebrew University, Jerusalem, Israel; 6 University of Turin, Department of Medical Science, Turin, Italy; 7 University of Algarve, Department of Biomedical Sciences and Medicine, Algarve Biomedical Center, Portugal; 8 Comprehensive Health Research Centre (CHRC), Portugal; 9 University of Naples “Federico II”, Department of Translational Science, Naples, Italy; 10 Psycho-oncology Unit, ANT Foundation, Bologna, Italy; 11 University of Verona, Department of Neurosciences, Biomedicine and Movement Sciences, Verona, Italy; 12 University of Naples “Federico II”, Department of Public Health, Naples, Italy

**Keywords:** frailty, community care, older people

## Abstract

The meeting of the European Innovation Partnership on Active and Healthy Ageing (EIPonAHA) action group A3 together with members of the Reference site collaborative network (RSCN) in December 2019 in Rome focused on integration of evidence-based approaches on health and care delivery for older citizens at different levels of needs with expertise coming from stakeholder across Europe. It was the final aim of the group to co-create culturally sensitive pathways and facilitate co-ownership for further implementation of the pathways in different care systems across Europe.

The study design is a mixed method approach. Based on data analysis from a cohort of community-dwelling over-65 citizens in the framework of a longitudinal observational study in Rome, which included health, social and functional capacity data, three personas profiles were developed: the pre-frail, the frail and the very frail personas. Based on these data, experts were asked to co-create care pathways due to evidence and eminence during a workshop and included into a final report.

All working groups agreed on a common understanding that integration of care means person-centered integration of health and social care, longitudinally provided across primary and secondary health care including citizens’ individual social, economic and human resources.

Elements for consideration during care for pre-frail people are loneliness and social isolation, which, lead to limitation of physical autonomy in the light of reduced access to social support. Frail people need adaption of environmental structures and, again, social resource allocation to maintain at home. Very frail are generally vulnerable patients with complex needs. Most of them remain at home because of a strong individual social support and integrated health care delivery.

The approach described in this publication may represent a first approach to scaling-up care delivery in a person-centered approach.

## I. INTRODUCTION

The understanding of health as a social continuum over the whole life span requires complex interactions within health and care systems to facilitate health and wellbeing for as many citizens as possible ^1^. Health and social domains are naturally affected by individual and community life environment, which do not always address older adults’ capacities during the trajectories of physical and cognitive functioning.

Therefore, an innovative response to individual citizens’ needs requires the implementation of actions at individual, population and system level, that demands political leadership and stronger commitment of authorities also making use of information and communication technologies ^2^. To enable this “transformation” it seems mandatory to evaluate the needs of citizens according to disease and functional capacities and to give access to this information to a broader audience ^3^.

Therefore, the aim of the 2019 winter meeting held in Rome by the European Innovation Partnership on Active and Healthy Ageing (EIPonAHA) - A3 action group and the reference sites collaborative network (RSCN) of the EIPonAHA was to prioritize the multidimensional assessment of frailty as the starting point for building up specific needs’ profiles of older citizens in primary and community care and to facilitate future health and social care scaling-up actions within the reference sites of the RSCN.

## II. METHODOLOGY

**Fig. 1:**
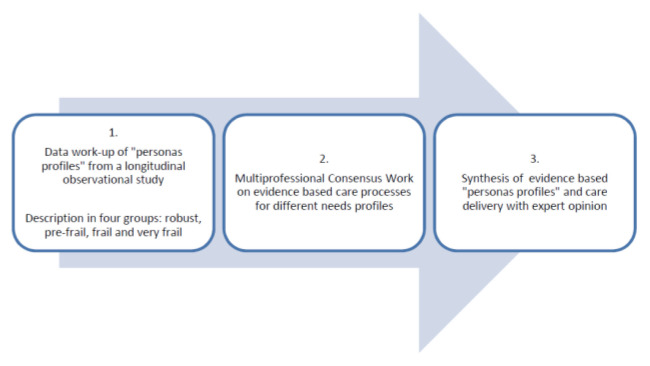
Methodological approach to define users’ needs profile for community and primary care Fig. 1 illustrates the methodological approach to elaborate users’ profiles and care needs adapted to individual functional capacity in a mixed method approach.

Given the nature of the topic, organizers chose a mixed method approach, combining data from literature with a qualitative consensus methodology ^4^ (Fig 1).

The work presented in this publication was divided into three steps: a data based preparatory work for personas profiles to ensure real-life setting, an open consultation process among experts and a final workup and transcription of results.

### A. Preparatory real-life data work-up of “personas profiles”

From 2014 to 2017 the Biomedicine and Prevention Dept of the Tor Vergata University Rome had collected personal data in a cohort of community-dwelling over-65 citizens, who were representative of Lazio region residents (Italy) in the framework of a longitudinal observational study. Additional to medical primary care, all the participants were evaluated in five domains (physical status, mental status, social resources, economic resources and functional status ^5^) by the administration of the Functional Geriatric Evaluation (FGE) questionnaire . The impairment in more than two domains identifies the individuals whose independency is threatened without provision of care services. This approach allows to cluster the population in four groups: robust, pre-frail, frail and very frail ^6^. In a next step, each cluster profile was described according to the following variables: demographics, diseases and comorbidities, capacity in performing ADL and IADL ^7^ and capacities for each of the five FGE domains. Based on the results of this description (see supplemental materials), three groups of experts of EIPonAHA and RSCN discussed issues of impact from the point of care delivery for the pre-frail, frail and very frail clusters^8^ (personas profiles). Data were stored in the database of the University of Rome Tor Vergata and analyzed using the SPSS program^9^.

Data used were collected in anonymized fashion meeting data protection rules (GDPR) as foreseen by the European commission ^6^

### B. Workshop

To achieve an open consultation consensus a structured and interactive workshop with representatives of regional stakeholders, partners from the A3 Action group of EIPonAHA and RSCN was organized. Groups were formed according to the three personas profiles. Each group included a multi-professional team, citizens and end-users, as well as Information Communication Technology (ICT) experts. It was the aim to achieve consensual results in the shape of interdisciplinary thinking corners focusing on matching well evaluated care solutions with the personas profiles and to detect barriers and facilitators for uptake of these solutions using the method of participatory learning and using a standardized approach ^10^.

Results of the working groups were summarized and transcribed using the method of Philip Mayring ^11^ and prepared for reporting by a core group also authoring this publication.

## III. RESULTS

### A. Results from search work

In total data from 1,334 community-dwelling older people attending the Tor Vergata University were evaluated cross-sectional and categorized as described. Detailed analysis data are shown in the supplementary material of this article. 42.6 % were detected as “robust”, 36.1% as “pre-frail”, 13.6% as frail and 7.9% as “very frail” according to criteria of selection outlined in the method section (2.1.). The four groups showed very different incidence of deaths and use of hospital services, including acute hospital admission rate and rate of accesses to emergency room, in the following three years, as expected and reported in a previous publication ^5^.

### B. Personas profiles

**Fig. 2:**
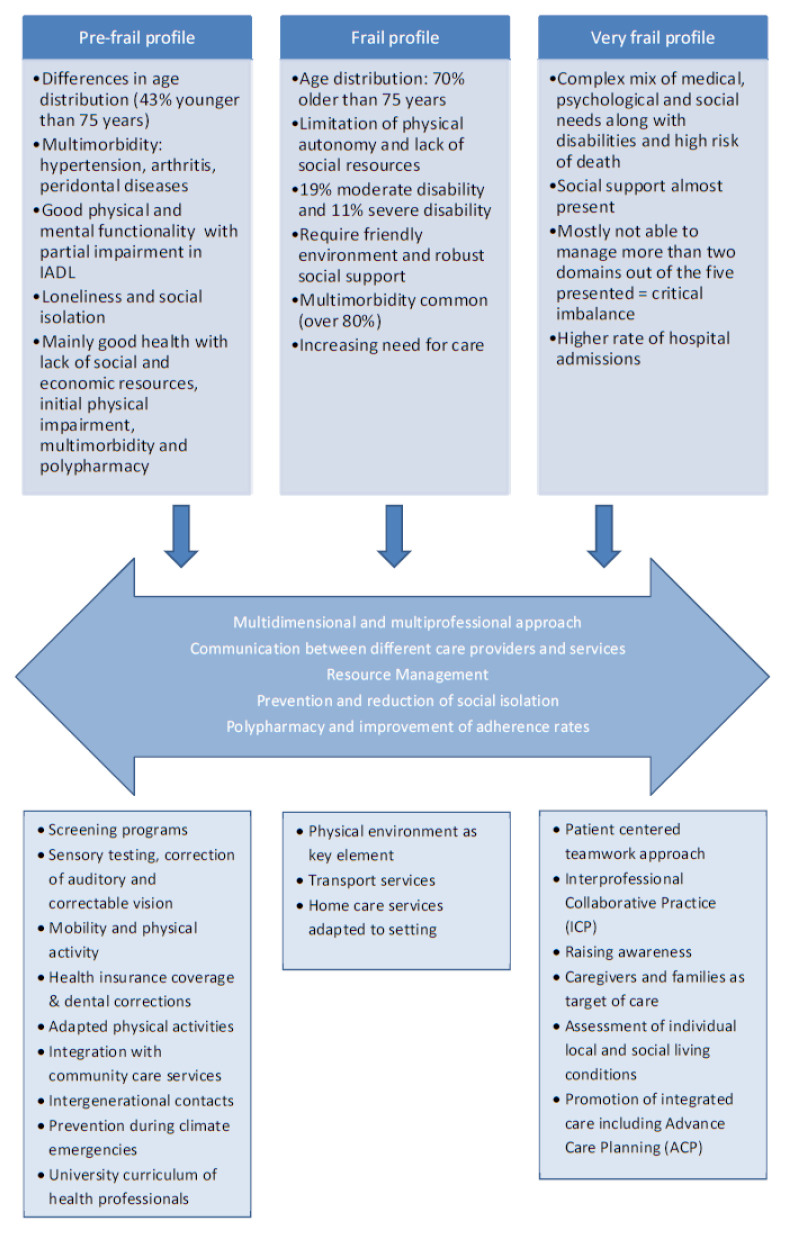
Personas profiles developed based on real life data from a community cohort of citizens in Rome older than 65 years Fig. 2 shows needs’ profiles detected through data analysis of community-dwelling citizens in Rome older than 65 years. As may be seen it was possible to develop users’ profiles for care and health care delivery based on health, functional and socioeconomic data. Older citizens were randomized as fit (not shown in this fig.), pre-frail, frail and very frail. Each group was further characterized due to individual needs from health and social care.

Based on cluster analysis it was possible to evaluate individualized needs’ profiles within pre-frail, frail and very frail citizens in the cohort. Figure 2 shows a summary of criteria which were commonly shared within patients in the three groups.

The persons defined as pre-frail were generally well with no significant difference in gender, but difference in age distribution, with 43% younger than 75 years. Many of the pre-frail people already present with multimorbidity, however, in general good physical and mental function; a small number of pre-frail people exhibiting moderate disabilities in Instrumental Activities of Daily Living (IADL). Only about 10% had mild cognitive impairment (MCI). From the psychosocial perspective, loneliness and social isolation were rather common: about 40% lived alone and close to 10% had no one to count on for social support.

In the frail personas group multimorbidity is very common (more than 80%). Frail personas were commonly found in the cohort older than 75 years (70% of frail people in the Tor Vergata population), with significant limitation in physical autonomy. Informal and formal care support was present in many of those frail older people, as otherwise “living at home” would have already been impossible for them. Interestingly close to 40% reported of still living alone and social care was provided by part time support. About 11% claimed of having nobody who can bring food at home in case of need and about 20% did not have anyone on which they can count on.

Very frail older citizens were generally vulnerable patients with complex health and social needs: in fact, they presented with a complex mix of medical, psychological and social needs along with disabilities and a high risk of death. High level of psycho-physical impairment (frequently due to neurological disorders) is associated to frequent severe disability (more than 68% of the individuals). The social support was present (otherwise to remain at home would have been impossible for them) even if many of them (close to 40%) were still living alone. Almost all of very frail were not able to manage more than two domains out of the five explored by the multidimensional evaluation with the need of immediate care interventions.

In the Tor Vergata Cohort the hospital admission rate was very similar between frail and very frail patients (384.7‰ per year and 392‰ per year for frails and very frail respectively). Cumulative access to hospital services was even higher in frail patients compared with very frail participants (1191.1‰ vs 848.4‰; p<0.001)^6, 12^.

### C. Results from Workshops on needs according to the social health determinants model [1]

In total 58 participants attended the two days meeting. All groups elaborating the needs of people included different professions as shown in table 1.

The spectrum of expertise present covered medical doctors, academic nurses, psychologist, partners from civil services, architects, nutrition experts, cooks, social workers, IT experts and experts in ambient assisted living research.

### D. Needs’ profiles of pre-frail, frail and very frail people according to social health determinants in an ecosystem of community and primary care

Figure 2 includes information on general recommendations covering care needs for the three different types of personas profiles in community as well as domains detected with special focus on the profiles. As may be seen domains for action included factors on microlevel as well as on meso and macrolevel of systems. **Food and nutritional supply, polypharmacy, and tailored pharmaceutical management** according to advanced care planning were detected as key elements for complex care also in community based older patients. The process of deprescribing should follow a patient-centered and team-based approach involving both the healthcare professionals and the patient/caregivers to effectively reduce inappropriate medication use. Participants detected polypharmacy and **improvement adherence rates** as one of the major challenges for the care of all older people in community.

Furthermore, there is bulk of evidence for the preventive effect of **physical activity**. Scaling-up of Adapted Physical Activities (AFA) is recognized as most effective to prevent or slow down physical decline ^13, 14^. Higher level of integration with community care services can allow larger publicity and increase participation in all those activities.

Research has linked **social isolation and loneliness** to higher risks for a variety of physical and mental conditions ^15^. Studies indicate also that **information and communication technologies (ICT)** have the potential to alleviate social isolation via various mechanisms, for example connecting to the outside world, gaining social support and engaging in activities of interests ^16^. Participants agreed that interventions aiming to prevent or reduce social isolation must become a focus area for policy and practice based on experimented models already available. Integrating swift communication between professions taking advantage of new ICTs seems a cornerstone to facilitate a person-centered care approach of citizens with complex care needs as well as those vulnerable to become frail. In this context the **role of case management** has to be designated to relatives (adequately supported by formal caregivers in case of need) and less frequently to professionals in order to integrate the services delivered to the clients in a person-centered and longitudinal manner.

However, in societies where living alone is often the case, resource management becomes crucial to close the gap between care needs and available support. The success of each intervention is determined by the integration with the client’s resources through an Individualized Care Plan (ICP).

### E. Special considerations for different cohorts of older people in community

Figure 2 also highlights recommendations synthesized during consultation work of the EIPonAHA. As may be seen experts reached consensus that implementation of regular screening programs, already in place in several countries, is recommended across Europe to detect and treat first cumulative deficits during community chronic care. ^17^. Based on these considerations, a package of prevention services can be developed and offered to older pre-frail adults in order to prevent or mitigate physical decline. In this framework, voluntary community support groups or patient associations were discussed as highly beneficial due to experience of participating stakeholders.

Given the rather complex care needs of frail older people, experts decided to adopt recommendations developed by the prefrail expert group and to expand to the frail group. However, being more homebound and equipped with less mobility, additional recommendations were developed for frail older persons: **Physical environment** was detected as key element to prevent adverse clinical outcomes for frail older people. The narrative “housing” was developed to describe a safe and personalized environment also through adapted architecture and design including ICT solutions (also see figure 2), where **risk of falls** is minimized. In many cases frail people need health care services, as well as meals or components/grocery goods delivered at home that are not always easily reachable ^17^. **Transport services accessible to this population** appear crucial to ensure them adequate care and, in general, the possibility to participate to social life. Integration between health and social services and with primary care should involve mainly epidemiologists, architects, ICT personnel and policy makers in order to ensure a stock of protected houses adequate to the frail population in need of this kind of services. **Multi-professional working groups** should be promoted to elaborate proposal to fit in specific life environments ^18,19^.

Building on results from the two other working groups, experts in care for very frail older people elaborated another two domains of interest to facilitate quality of care for those older people, highly in need for support and medical integrated care. Many studies suggest that a patient centered teamwork approach is essential for constructing a high quality, safe and reliable health care delivery system ^20^. In this context, a capability framework of core competences for caring frail older people has been developed by experts and was adopted during this meeting^21^. Aiming towards Interprofessional Collaborative Practice (ICP) for care of very frail older people, a cultural change is needed and the group agreed that guidelines should be drafted to facilitate implementation and scaling-up processes within European Member States (MSs).

Furthermore, care for very frail older people widens the focus towards **caregivers and families** as the target of the care. Literature provides an abundance of studies about challenges encountered by family caregivers, all of which demonstrate that caregiving activity has an impact on physical and mental well-being and informal care is largely provided by females without caregivers’ education and training and continuous feedback and support ^22, 23^. The group assumed that helping caregivers is one of the most cost-effective long-term care investments we can make.

To facilitate the achievement of an optimal care of very frail older people the group recommended two key actions to be implemented across all care settings within European regions: assessment of individual local and social living conditions of affected older people on a regular basis and promotion of integrated care, including Advance Care Planning (ACP). ACP is a process where a person discusses values and healthcare preferences with their family and healthcare team. The goal is to help a person make informed decisions for their future medical treatment. This approach allows re-thinking of therapeutic goals thereby tailoring medical treatments from “cure” to “care and supportive” medical action in an integrated and longitudinal fashion.

## IV. DISCUSSION

Major challenge for many countries is the integration of community-based models of care for older citizens across a wide range of functional capacities. Data show that the top-down approach for such integration processes has not yet achieved the promotion of a systematic approach to integrated care almost everywhere in Europe ^24^. Some success stories are available and they can provide interesting insights about factors associated to their results ^25^. The EIPonAHA A3 Action Group together with the RSCN has focused its works on this challenge over the past years ^26^.

The strength of the consortium of the EIPonAHA resides in its capacity in public health, as well as in multidisciplinary and clinical competence. In this context key deliverables such as overviews on frailty as a public health concept ^14^ and frameworks such as a new food and nutrition model, the “NutriLive Approach” ^27^ have been launched previously.

In the current publication, authors describe how real-life data from community-dwelling older citizens may be used to co-create (care) recommendations in a mixed-model approach ^4^. This approach is unique in different ways. First, the development of personas profiles is based upon real life data. It is interesting to note that general shared recommendations were developed for all three personas with functional limitations. These recommendations mainly addressed system-based factors like communication pathways for cure and care of older citizens, resource management with a special focus on social care and prevention from isolation as major trigger for progression of loss of functional capacity. A single-professionalism approach that takes leadership and leads others is doomed to failure in a society where the variables who contribute to the change of life-style are so many and so different and the demand of care is strongly individualized. Therefore, the more we are able to include different professionals in this process, the more we will be able to experiment this model and its impact on the quality of life of the European older citizens.

Over the last 20 years, Europe has been looking for a model of Long-Term Care (LTC) best suited to respond to the ongoing welfare transition. However, many of those models seem to have failed to solve the care demand rising due to the current demographic shift in Europe ^28^. Translating care responsibilities to community requires not so much a single “European” model but flexible solutions that can be “customized” to each country and the personalized needs of ageing citizens. Indeed, it is possible to identify common elements to all the countries that constitute a shareable denominator from which to start to improve community care for older citizens. A Bio-Psycho-Social frailty model is one of these elements, a synthetic indicator of welfare demand, associated with the consumption of socio-health resources and also disability, dependence and mortality. Starting from screening for functionality, followed by the assessment of frailty as a multidimensional quantity combining elements of psycho-physical health, functional capacity and socio-economic resources, it is possible to design and monitor integrated social and health care interventions, that positively affect survival and quality of life of senior citizens. However, the awareness of the potential effectiveness of the care approach based on the assessment of frailty is not yet sufficiently supported by scientific evidence, so as to limit its diffusion.

To overcome the obstacles to integrated care a crucial role is played by training and education of different professionals to operate jointly at community level, where the need for integrated knowledge and combined performances is fundamental ^29^. Beyond the internal circle of health and care, it seems necessary to include structural, social and environmental factors to facilitate wellbeing and care at home for many older people.

The perspectives presented in this publication try to facilitate future discussion among stakeholders and support evidence-based political decision making ^30^.

## V. CONCLUSION

The current study describes work done by stakeholders from different professions and affiliated to the mentioned partnerships, putting together proposals of service integration for distinguished personas groups using a mixed-model bottom up approach. Experts agreed on the rationale for a multidimensional view in the care for older people building on interprofessional collaboration. This system-based agenda built by the partnership and presented here clearly reflects a call for action to rebuild health and social care systems to facilitate ageing well across Europe.

## Figures and Tables

**Table 1:** Participants in different experts’ groups at the EIPonAHA workshop tailoring needs for personas from pre-frail to frail and very frail and living in community Table 1 shows the distribution of professional capacities presented within the experts’ groups in the workshop in Rome in 2019. Distribution of different professional capacities was comparable among the three groups elaborating needs profiles according to the personas approach [2].

	MDs (Medical Doctor: Geriatrician, Cardiologist, Epidemiologist, Hygienist)	RNs (Registered Nurse)	SWs (Social Workers)	Others*	Total
Pre-frail group	6	4	1	11	22
Frail group	9	4	1	9	23
Very frail group	6	1	1	5	13
Total	21	9	3	25	58

* (Biologists, economists, statistics, sport science, mechanical engineer, psychologist, architects, ICT experts and volunteers)
